# Endotracheal Intubation in a Neonate with Esophageal Atresia and Trachea-Esophageal Fistula: Pitfalls and Techniques

**Published:** 2014-04-01

**Authors:** Bharti Taneja, Kirti N Saxena

**Affiliations:** Department of Anesthesiology, Maulana Azad Medical College, New Delhi-110002, India

## INTRODUCTION

The insertion of an endotracheal tube (ETT) in a patient of esophageal atresia (EA) with trachea-esophageal fistula (TEF) can be challenging for even the most experienced of anesthesiologist. Incorrect placement, if not detected on time, can have the most devastating complications. The correct placement of an endotracheal tube is dependent on the anatomy and morphology of the fistula. Holzki et al [1] in their study of bronchoscopy on 113 patients of EA with TEF reported that in 11% of patients, the fistula was below the level of carina, in 22% the fistula was within 1cm of the carina while in 67% the fistula was 1cm above the carina. Furthermore, these patients sometimes have other associated congenital anomalies such as tracheomalacia and vascular ring that require additional intervention.

The anesthetic and surgical management of neonates with EA with TEF focuses on ventilation of the lungs while avoiding ventilation through the fistula. The mainstay of airway management focuses on careful tracheal intubation beyond the fistula with avoidance of muscle relaxants and positive pressure ventilation until the fistula has been ligated. Gastrostomy, either preoperatively under local anesthesia or soon after the induction of general anesthesia is sometimes used to decompress the stomach and prevent gastric distention.[2]

Special attention to placement of the endotracheal tube in the trachea is warranted. The incorrect placement of an ETT can cause increased morbidity by ineffective ventilation as a result of migration of ETT in the fistula. The additional gastric distension would lead to an increased intra-abdominal pressure and potentially cause decreased venous return with hemodynamic compromise as well as restriction of diaphragmatic excursion with significant respiratory embarrassment. Gastric distension can also result in the aspiration of gastric contents via the TEF causing aspiration pneumonitis. Aspiration pneumonitis alone has accounted for up to 50% of the perioperative morbidity and mortality in this patient population.[3] Thus theoretically, the sooner the TEF is closed, the less likely are any of the predictable complications.

**Techniques of Endotracheal Tube Placement:**

The ideal method of intubation is placement of the tracheal tube tip beyond the fistula. This facilitates adequate ventilation and prevents gastric insufflation. However, this is not always possible especially in large low lying fistulas. Various techniques have been advocated for accurate endotracheal tube placement and depend on the type and site of fistula, pulmonary status and co-morbid conditions. Direct laryngoscopy or auscultation may not be confirmatory for correct placement of the ETT. Also, a properly placed ETT at the beginning of the case may slip into a large fistula. This can happen most frequently during lateral positioning of the patient for thoracotomy or thoracoscopy.[4]

**Role of Bronchoscopy:**

Preoperative bronchoscopy may help the anaesthesiologist and the surgeon to assess the size and location of the TEF and plan for the best airway management strategy. It provides information about the exact size of the fistula, its location, and other coexisting airway anomalies, including the presence of proximal fistula.[1,4,5] Additionally, it is also be useful to confirm ETT placement after endotracheal intubation and intra-operatively in the event of any cardio-respiratory instability or ETT dislodgement. The routine use of preoperative bronchoscopy by Aztori et al modified the surgical approach and management of 24% patients.[4] 

Most tertiary care centres in the west advocate the use of either a rigid or flexible bronchoscope for the identifying of the site and anatomy of the fistula as well as for guiding endotracheal tube placement.[6] Flexible bronchoscopy can be used to confirm proper placement of the ETT and rule out any migration of the endotracheal tube with position changes. In the event of intra-operative tube migration and subsequent hypoxia urgent bronchoscopic evaluation and re-positioning of the ETT is advocated even during thoracotomy. The rigid bronchoscope offers the possibility to ventilate the patient while also visualizing the airway. Other adjuncts in case of continued cardiorespiratory instability include needle decompression of the stomach and pleura as well as occlusion of the fistula with a balloon via emergent gastrostomy or via bronchoscopy.[7-10]

Considering the benefits conferred by bronchoscopy and given the wide-spread availability of this equipment, it should be considered standard practice in all patients of EA with TEF.[5,11]

**Minimal Invasive Repair of TEF:**

Minimally invasive surgical techniques are being used increasingly for the repair of EA with TEF in neonates. Traditional repair through a right dorsolateral thoracotomy has major disadvantages, including a large scar, significant postoperative pain, and a high degree of scoliosis and shoulder girdle weakness later in development. An advantage of minimally invasive surgery is superior visualization of the fistula and the surrounding anatomy through the thoracoscope. However, this new approach poses extra challenges to the anesthesiologist because of the requirement for collapsing a lung during the thorascopic repair. Thus, the anesthesiologist needs to isolate not just the TEF, but also a mainstem bronchus during the procedure.

**Airway management and placement of ETT:**

The correct placement of ETT below the level of fistula is crucial during endotracheal intubation and prior to ventilation. Therefore, spontaneous respiration is maintained during induction with inhalational anaesthetic agents and muscle relaxants are administered only after the ligation of the fistula so as to prevent gastric insufflations from positive pressure ventilation. Alternatively, a rapid sequence intravenous induction without positive pressure mask ventilation may be the preferred mode of induction followed by rapid securing of the airway.[12] Several different methods for securing the airway and controlling the fistula have been described. 

**Traditional technique:**

The technique most commonly used involves insertion of the ETT deep endobronchial followed by gradual withdrawal till the ETT is just above the carina and bilateral equal breath sounds are heard on auscultation of the chest.[2] Additionally, the ETT is rotated so the bevel faces anteriorly and away from the fistula which is most commonly located on the posterior wall.[10] In this manner, the tip of the ETT is placed beyond the fistula and the anteriorly placed bevel ensures that the shaft of the endotracheal tube occludes the fistula. For obvious reasons, an ETT without side holes is recommended. The correct placement maybe confirmed either by auscultation or by bronchoscopic evaluation. In case a gastrostomy has been performed previously, the end of the gastrostomy tube may be placed underneath a water seal. The presence of bubbling indicates ventilation through the fistula, which will occur if the tip of the ETT is proximal to the opening of the fistula. The ETT should be pulled back almost until gas begins to bubble from the end of the gastric tube (which has been placed under water seal), then re-advanced until the bubbling stops.[2] A capnograph inserted into the gastrostomy tube will indicate the same thing by showing tracing of respiratory movements and persistently elevated levels of end tidal carbon dioxide levels.[13]

This technique is especially useful in emergency situations and when TEF is associated with duodenal atresia.

**Use of Fogarty Balloon Catheter:**

Use of Fogarty balloon catheter to occlude the fistula is another method commonly employed. After the induction of general anaesthesia, a suitable sized Fogarty arterial embolectomy catheter is placed through the vocal cords under direct laryngoscopy and then the bronchoscope is placed through the cords. The Fogarty balloon-tipped catheter is then advanced into the TEF. The Fogarty catheter often preferentially passes into the TEF because of the dependent position of the TEF. The fistula is then occluded by inflating the balloon-tipped catheter. The bronchoscope is removed, and the trachea is intubated with an oral ETT in the standard fashion and the ETT is placed in the trachea alongside the Fogarty catheter.[9] The insertion of the Fogarty catheter can be from either the tracheal route (2-3Fr) or via the gastrostomy (5Fr).[14]

However, apart from being technically complex, this technique has its own problems. The occlusion of the fistula with a Fogarty embolization catheter may or may not be effective. In order to place the Fogarty catheter with a rigid bronchoscope, it would be required to interrupt ventilation and the size of the bronchoscope may limit use of the catheter. In the event of the Fogarty catheter getting dislodged intra-operatively, it may occlude the trachea making ventilation impossible. Also, the catheter can damage the esophageal mucosa at the balloon site.[10] Further, gastrostomy is not routinely performed in patients with no other complications, so, retrograde occlusion through a gastrostomy may not always be an option.[15] 

Despite the above mentioned drawbacks, Fogarty catheter is a useful aid in isolating large fistulas or those located near the carina.

**Use of Double Fogarty Catheters:**

Placing two balloon-tipped blockers, one in the fistula and the other in the right mainstem bronchus, is a viable technique for thoracoscopic EA with TEF repair when the fistula is at or very close to the level of the carina and one lung ventilation is required. In a case report [16] on a full-term neonate with a type C type EA with TEF (fistula at the level of the carina), two Fogarty type balloon-tipped embolectomy catheters were placed alongside the ETT to successfully achieve the goal of blocking ventilation of the fistula and the right lung. The balloon of one catheter blocked the fistula while the other was inserted into the right mainstream bronchus to occlude the ventilation of the right lung. The use of fibreoptic bronchoscopy greatly facilitated placement of the blockers. The patient made an uneventful recovery.[16]

The disadvantages of bronchial blockers include the possibility of mucosal damage (though not yet seen), retrograde migration of either blocker into the tracheal lumen, resulting in partial or complete airway obstruction. The insufficient blockade of the mainstream bronchus may lead to partial ventilation of the collapsed lung and bronchial rupture.[17]

**Use of Cuffed ETT:**

The cuff of an ETT can be similarly used to block the fistula. Immediately following the insertion of the ETT, a 2.5 mm flexible bronchoscope is inserted into the ETT. The ETT is advanced under direct vision to just above the carina but distal to the fistula. At this location, the cuff on the ETT is inflated to occlude the fistula. Greenberg et al have reported accurate placement of a 3.5mm cuffed ETT with bilaterally equal breath sounds on auscultation and without gastric distension on positive pressure ventilation.[18] Thus, a cuffed ETT may be accurately positioned to exclude the fistula allowing for an easier and safer operation.[12]

**One lung Ventilation:**

Many case reports have documented the successful use of one-lung ventilation in term and preterm neonates. After the administration of general anaesthesia, the ETT is facilitated into the left mainstream bronchus till the ligation of the fistula after which it is slowly withdrawn back into the trachea for oesophageal anastomosis. This technique of inserting the ETT into the left mainstream bronchus acts by blocking the fistula and the right mainstream bronchus simultaneously.[19,20] However, differences in the diameters of main-stem bronchus and the trachea may result in an ETT that fits a main-stem bronchus well but is too small for the trachea while the one that fits the latter is too large for the former. This might predispose to left bronchial edema.[21] In one report, left upper lobe collapse was also seen in deliberate endobronchial ETT placement in neonates to achieve one-lung ventilation.[19] Another disadvantage is occurrence of desaturation during one-lung ventilation in EA with TEF repair.[12] The ETT may need to be retracted several times to ventilate both lungs intra-operatively and subsequent repositioning of the ETT tip back into the left mainstream bronchus requires fibreoptic bronchoscopy, which may be hazardous and cumbersome in a semi-prone neonate, made even more difficult by the sterile drapes separating the small distance between the operative field and the patient’s mouth.

Use of a specially designed bifurcated tracheal tube for EA with TEF repair has also been described.[22] This is not a double-lumen tube, and is therefore not suitable for differential lung ventilation.

**Use of Endobronchial blocker:**

In neonates with EA and a large carinal TEF, endobronchial blocker has been used to occlude the fistula.[23] The endobronchial blocker is inserted down to the level of the mid-trachea and the proximal trachea was intubated with a microcuff endotracheal tube alongside the blocker, while, the fiberoptic bronchoscope is used to guide the blocker into the fistula. Use of a new 5-Fr endobronchial blocker suitable for use in children with a multiport adapter and fiberoptic bronchoscope has been described.[24] This helps reduce the incidence o hypoxemia and also aids repositioning of the endobronchial blocker intra-operatively.

## CONCLUSION

The airway management and ETT positioning in a case of EA with TEF is definitely challenging but is aided to a great extent by preoperative and intra-operative bronchoscopy. Out of the various techniques mentioned above, the traditional technique of endobronchial intubation followed by gradual withdrawal into the trachea remains the most popular amongst anaesthesiologists. The technique of choice must take into account the type and location of fistula, the preoperative chest condition, pulmonary compliance and other associated co-morbid conditions. The positioning of the ETT in the left mainstream bronchus or distal to the fistula may minimize gastric insufflation and improve ventilation as well as surgical field, however, the surgeon and anaesthesiologist must remain vigilant for any inadvertent tube mal-positioning with its catastrophic sequelae at all times.

## Footnotes

**Source of Support:** Nil

**Conflict of Interest:** None

**Editorial Comment:** Many pediatric surgeons would disagree with the above recommendation of the authors to place the ETT tip beyond the TEF. Instead, if the tip of ETT is placed just above the TEF, it is easier to identify the lower esophageal segment in the most common variant of EA- type C with distal TEF; it would distend with each breath. However, there is no randomized trial or metanalysis available to suggest the superiority of one over the other. 

